# Using Human Disease Outbreaks as a Guide to Multilevel Ecosystem Interventions

**DOI:** 10.1289/ehp.7122

**Published:** 2004-05-27

**Authors:** Angus Cook, Andrew Jardine, Philip Weinstein

**Affiliations:** School of Population Health, University of Western Australia, Perth, Australia

**Keywords:** bioindicators, disease control, disease outbreaks, ecologic management, ecosystem health, surveillance

## Abstract

Human health often depends on environmental variables and is generally subject to widespread and comprehensive surveillance. Compared with other available measures of ecosystem health, human disease incidence may be one of the most useful and practical bioindicators for the often elusive gauge of ecologic well-being. We argue that many subtle ecosystem disruptions are often identified only as a result of detailed epidemiologic investigations after an anomalous increase in human disease incidence detected by routine surveillance mechanisms. Incidence rates for vector-mediated diseases (e.g., arboviral illnesses) and direct zoonoses (e.g., hantaviruses) are particularly appropriate as bioindicators to identify underlying ecosystem disturbances. Outbreak data not only have the potential to act as a pivotal warning system for ecosystem disruption, but may also be used to identify interventions for the preservation of ecologic health. With this approach, appropriate ecologically based strategies for remediation can be introduced at an earlier stage than would be possible based solely on environmental monitoring, thereby reducing the level of “ecosystem distress” as well as resultant disease burden in humans. This concept is discussed using local, regional, and global examples, thereby introducing the concept of multilevel ecosystem interventions.

During the construction of the Panama Canal in the 1880s, continuous outbreaks of yellow fever killed > 5,500 workers, or > 6% of the workforce. The most immediate cause of those outbreaks was, then as now, obvious and was captured in the records of artist-to-be Paul Gauguin, who was then a digger with the French Canal Company: “At night I am devoured by mosquitoes” (Harrison 1978). The ultimate cause of these outbreaks was more complex, however, involving disruptions to both environment and society, mediated by a range of political and economic drivers.

Models have been developed to describe the way in which such interacting disruptions influence health (Parkes and Weinstein 2004), but from a biophysical perspective one of the more constructive lines of analysis is directed toward the disruption of the immediate ecosystem. During the Panama Canal construction, it is obvious (with the wisdom of ecologic hindsight) that replacing a rainforest environment with an urban/industrial environment offers the opportunity for container-breeding vector mosquitoes to proliferate and to transmit disease at a scale never before encountered in an affected area. By today’s standards, this disease outbreak and the associated ecosystem disturbance might seem to have followed a relatively obvious path. Nevertheless, despite a greater contemporary understanding of microbiologic and ecologic dynamics, insults to the environment occurring even in modern times are often discovered only as a result of detailed outbreak investigation.

Measurable bioindicators of ecosystem health were first described in detail by Rapport et al. (1985). These include changes in nutrient cycling, decreased species diversity as a result of decreasing habitat diversity, retrogression (a reversal of the normal process of species succession as the ecologic community is simplified), and increased fluctuations in population size. Presence of disease also explicitly formed one of the bioindicators, and it was suggested that increased disease incidence among plant, animal, and human populations would manifest as the fabric of the ecosystem begins to deteriorate and natural buffering and protective mechanisms break down.

The intrinsic link between ecosystem health and human disease (especially vector-mediated disease) has been discussed in a number of previous publications (Cassis 1998; Chivian 2001; Epstein 1995; Forget and Lebel 2001; Haines et al. 2000; McMichael 1997; Nielsen 2001; VanLeeuwen et al. 1999; Waltner-Toews 2001). These authors have noted that ecosystem health is heavily influenced by human activities and that, vice versa, human health depends on proper ecosystem functioning. Reflecting this close relationship, it has been suggested that disease incidence within a human population can be used as a bioindicator or “yardstick” of the health of the ecosystem of which the community is a part (Rapport 1999).

We concur with this concept and advance it two steps further by contending *a*) that human disease incidence is in fact one of the most useful and practical bioindicators of the health of an ecosystem and *b*) that using human health as a bioindicator in this way can assist in guiding rapid and appropriate ecosystem interventions. A major advantage in using disease outbreaks as bioindicators of even subtle ecosystem disruptions is that the health of human populations is generally subject to more widespread and accurate surveillance than is ecosystem health (Spiegel and Yassi 1997). Many sources of data, such as data obtained from disease registries, infectious disease notification systems, and hospitalizations, provide ongoing measurement and monitoring of human communities. It should be emphasized that we are not advocating that information on human suffering should simply be used to better preserve the environment. Our approach very much supports dual end points: early and appropriate minimization of ecologic degradation in its own right, with the major consequence that this is the pathway by which we will preserve the public health for communities living in these environments.

The incidence data most useful in signaling underlying ecosystem processes relate to vector-mediated diseases (e.g., arboviral illnesses), direct zoonoses (e.g., hantaviruses), and infections that appear to transcend simple transmission categories [e.g., viruses that were zoonotic but “transformed” to direct anthroponoses, such as SARS (severe acute respiratory virus) and HIV (human immunodeficiency virus)]. A number of direct anthroponoses (i.e., disease spread by direct human-to-human transmission, such as measles, polio, and chlamydia), reflect human dynamics such as crowding and sexual contact, so their roles as ecosystem bioindicators are less likely to be pertinent. However, ecologic disruption may act as an indirect or partial determinant even for some of these infections (e.g., as in the case of cholera transmission; Tauxe et al. 1994).

As we outline in the examples below, many stresses and disruptions to natural ecosystem functioning are identified only as a result of detailed epidemiologic investigations, which in turn follow an increase in human disease incidence detected by routine surveillance. By identifying ecosystem disruptions that affect human health using this outbreak-based approach, appropriate strategies for intervention and remediation can be introduced at an earlier stage than would be possible based solely on environmental monitoring. Although it is still possible to detect ecosystem disruption using traditional environmentally based bioindicators of ecosystem health, this generally requires a detailed and complex investigation in terms of the cost and feasibility of obtaining and analyzing valid and consistent data (Patil et al. 2001; Rapport et al. 1995). The interpretation of ecologic indicators obtained can also be problematic, and the results may be difficult to convey to policy makers and a broader public (Schaeffer 1996). To illustrate our argument, we discuss examples of disease outbreaks that have led to the identification of ecosystem disruptions and the appropriate corresponding ecosystem interventions at local, regional, and global levels using the comprehensive framework provided by the Millennium Ecosystem Assessment (2003) as a basis.

## Ecosystem Interventions for Local Outbreaks

The paradigm that draws together human outbreaks (as identified by, e.g., disease surveillance data) and environmental disruption will first be discussed in relation to local ecologic transformations. Among the best-documented examples is deforestation, which is often accompanied by ecologic simplification toward either monoculture or subsistence agriculture. The outbreaks of monkeypox in Zaire and hantaviruses in the Americas have both acted as clear bioindicators for disruption of the local distribution of natural vegetation. The clearance and replacement of complex rainforest, such as through slash-and-burn clearing practices, have encouraged a massive proliferation of small animals (e.g., rodents and squirrels) that act as vectors for both of these viral diseases (Glass et al. 2000; Khodakevich et al. 1988). Compared with the cases of human disease that arose as a result, few other routinely obtained and readily interpretable bioindicators were able to alert the authorities and general public to the extent of the underlying ecologic process. Ecosystem interventions that were readily suggested were to limit the removal of forest and impingement of human communities on the habitats of virus-carrying mammals.

Another example of the link between localized outbreaks as an indicator for ecosystem health is provided by Ross River virus (RRV) patterns in Australia. RRV is the most common arboviral infection in Australia—with 52,053 laboratory-diagnosed cases reported from when reporting began in 1992 until the end of 2003 (Communicable Diseases Network Australia 2004)—and is characterized by traditional rheumatic joint manifestations, rash and constitutional effects, and more recently described presentations including glomerulonephritis (Selden and Cameron 1996). Distinct seasonal epidemic activity is observed in northern tropical regions during summer months when rainfall is highest. In some areas virus activity may persist year-round, but winter rainfall in tropical regions is generally insufficient to support vector breeding and thus limits transmission during the dry season (Russell 2002).

This typical disease pattern in the tropical northeast Kimberley region of western Australia has undergone recent changes. Unusual dry-season cases of RRV disease led to suspicions that ongoing development of the Ord River Irrigation Area had disrupted the local natural ecosystem to the extent that mosquitoes were now able to breed year-round. A subsequent entomologic investigation during August (usually the driest month of the year) confirmed that mosquito breeding was indeed occurring in the dry season (Jardine et al., in press). Significantly larger numbers of adult and larval *Culex annulirostris*, an important vector of RRV and a range of other arboviruses in Australia, were collected within the irrigation area compared with nonirrigated reference areas.

These disruptions to the dry-season ecology of the mosquito fauna in the area were therefore detected only as a result of an investigation sparked by an unusual outbreak of human disease. Routine mosquito surveys, which might have acted as an alternative bioindicator, are simply not carried out in the dry season. These findings suggest that appropriate ecosystem strategies to reduce breeding of disease vector mosquitoes should focus primarily on restoring local hydrology to reduce potential mosquito breeding habitats. In particular, the ability of mosquitoes to breed year-round means control activities must be ongoing and not restricted to a few months during the peak of the wet season (Jardine et al., in press).

## Ecosystem Interventions for Regional Outbreaks

Illness patterns at a wider, regional level may also relate to and act as telltale signs for disturbances of usual ecologic processes. The recent epidemic of new variant form of Creutzfeldt–Jakob disease from beef consumption led to the identification of a little-recognized ecologic anomaly, with investigations into the source of the disease revealing that animals that are naturally herbivorous—beef cattle—were being transformed into carnivores through the introduction of meat and bone meal in their feed (Wilesmith et al. 1988, 1991, 1992). For regions that engage in such practices, the early intervention suggested was to reverse this food-chain anomaly by banning material extracted from other mammals in cattle feed (Nathanson et al. 1997).

A more complex food-chain disruption was highlighted by the increased incidence of Lyme disease in the northeastern United States during the last two decades. The “emergence” of this disease led to detailed ecologic studies of vector ticks in the genus *Ixodes*, which transmit the pathogen *Borrelia burgdorferi* from white-footed mice to humans. In a healthy ecosystem, a variety of small mammals are available for these ticks to feed on, and most of these hosts do not carry *Borrelia* spirochetes. Thus, the proportion of infected ticks is small, and the probability of *Borrelia* transmission to humans is low. However, with the growing disruption of regional ecosystems, the diversity of small mammals decreased and was replaced by burgeoning numbers of white-footed mice (LoGiudice et al. 2003). This mouse species, which multiplied and invaded the niches vacated by more sensitive animals, in turn became the more common host for the ticks. To complicate matters further, more mature ticks feed on deer, which have in turn proliferated because of the removal of major predators (wolves) from the food chain in these areas. The net result is a larger tick population with a higher percentage of infected reservoir species, all more likely to infect the increasing numbers of humans impinging on a once pristine regional ecosystem. An appropriate regional intervention suggested is restoration of biodiversity in such ecosystems, which would reduce the abnormal proliferation of white-footed mice, deer, ticks, and reservoirs for *Borrelia* (Wilson 2002).

The link between regional outbreaks and ecosystem change is further illustrated by the periodic emergence of ciguatera fish poisoning. Ciguatera, linked to toxic marine dinoflagellates (*Gambierdicus toxicus*), is a syndrome characterized by acute gastroenteritis and neurologic symptoms (including inverted temperature perception, an odd symptom whereby cold objects appear hot to touch and vice versa). The severity of poisoning ranges from imperceptibly mild to rapidly lethal, and there is generally a history of cases having consumed tropical reef fish. In Pacific Island countries that rely on fish as a major source of protein, ciguatera poisoning is the cause of a significant disease burden (Laurent et al. 1993), and anecdotal evidence suggested that this burden increased through the late 1990s. Although subsequent investigations demonstrated a correlation between sea surface temperature and the number of cases, particularly on islands strongly influenced by El Niño climatic conditions (Hales et al. 1999), dinoflagellate proliferation is probably most enhanced by physical disturbances to coral reef ecosystems. When reefs are blasted (e.g., for the coral trade) or suffocated (by runoff from deforested hillsides), massive coral death occurs, creating extensive substrates for the growth of macroalgae (Kohler and Kohler 1992). It is on the surface of these macroalgae that ciguatera-causing dinoflagellates grow, and damaged coral ecosystems may therefore be particularly productive of toxic fish. Hales et al. (1999) based their study into the underlying ecosystem disturbances accounting for ciguatera on data collected as part of routine health surveillance; no equivalent environmental monitoring data were available on the “health” of coral reef ecosystems around the Pacific. Again, the findings also suggested possible regional solutions, including remediation of selected coral reef ecosystems, such as by reforestation of hillsides to limit runoff and avoidance of blasting, especially on those islands where most ciguatera cases are occurring.

## Ecosystem Interventions for Global Outbreaks

In particular circumstances, the consequences of ecosystem impingement and disruption may become apparent on a global scale. One pathogen whose emergence, with devastating consequences, was driven partly by ecosystem distress is HIV/AIDS. This retrovirus has overwhelmed the communities of many countries, including those that were already highly socially and economically vulnerable (Tinker 1988). It took some years for the origins of the virus to become apparent, but most now believe that it originated from simian reservoirs: probably chimpanzees for HIV-1 and sooty mangabeys for HIV-2 (Gao et al. 1999). The stage of transferal to human populations most likely occurred with the practice of using primates as a food source. Indeed, before the disease expanded to such a devastating level, neither the scale nor implications of “bush meat” practices were fully acknowledged (Tutin 2000). Such dietary practices have a potential capacity to transmit other retroviruses, of which at least 20 simian forms have been identified (Dalgleish and Weiss 1999). Indeed, Wolfe et al. (2004) have confirmed zoonotic infections with simian foamy virus in residents of central African forests who reported direct contact with blood and body fluids of wild nonhuman primates.

The implications for preserving ecosystem health suggested by the example of the global HIV/AIDS outbreak are clear. One response to the threat is to reduce any further risk of simian retroviral transmission by responding to the ecologic disruptions that HIV/AIDS brought to light. However, to minimize the infective risk of simian retroviral infections to the general population, remedial measures must occur in those original environments from which emergence occurred: rainforests or other habitats in which primates thrive (Bisong 1999). In other words, the optimal ecosystem interventions required to limit retroviral spread are quite geographically remote from most of the global population even though they comprise the majority of people who would probably suffer the consequences of a further HIV-like outbreak. Most options to further reduce rainforest penetration in the remote parts of other continents would entail a considerable degree of operational complexity and impinge on the sovereign rights of countries to manage their own ecosystems (often in the face of profound poverty). Ecosystem health—and ultimately, human health—might be served only by simultaneously addressing the socioeconomic deprivation that drives forest clearances and consumption of primates and encouraging alternative sources of cropping and land management (Stephens et al. 2002).

The opportunity for multiple approaches to ecosystem intervention is clearly evident for the arboviral disease dengue fever. The occurrence of one or more of the dengue serotypes across most tropical regions of the world (Wilson and Chen 2002) reflects ecosystem disturbances at multiple levels in a manner that few traditional bioindicators could capture. Unlike the threat posed by simian retroviruses, which may respond to local actions to reduce bushmeat contact, multiple ecosystem interventions are suggested for dengue control that operate at numerous, often overlapping levels: locally, to remove artificial breeding habitats for the *Aedes* mosquito vectors that are provided by containers (Knudsen 1995; Moore et al. 1990; Tauil 2001); regionally, to limit the disruption of waterways (which encourage stagnation and high nutrient loads) (Forattini et al. 2001); and globally, to minimize the effects of global warming that encourages mosquito breeding for longer durations at a wider range of latitudes (Chan et al. 1999; Hales et al. 2002; Hopp and Foley 2003). The climatic instability associated with the warming trend may also drive excess rainfall and flooding in many areas, thus again providing ideal breeding sites. Thus, information about outbreaks of dengue fever, reliably monitored in many countries, can inform an integrated approach—operating at three levels—to the management ecosystem disturbances.

## Discussion

We live in an era of emerging and reemerging infectious disease attributable to ecosystem disruptions (Weinhold 2004), a phase that has been termed the third epidemiologic transition (Barrett et al. 1998; McMichael 1993). Given the threats to health in this modern milieu, understanding and assessing the links between anthropogenic pressure on ecosystems, human health, and ecosystem structure and functioning are vitally important (Koren and Crawford-Brown 2004). Although currently “there is no simple solution to a quantitative and quick assessment of ecosystem health” (Ramade 1995), we contend that human disease surveillance (particularly notification systems for infectious disease) at local, regional, and global levels is often a readily available and accurately recorded bioindicator that could be used for such purposes. Monitoring of disease events is more widespread, accurate, and subject to ongoing quality assurance than many of the “indicators of ecosystem health” that have been proposed in the past (Spiegel and Yassi 1997), which are often difficult to routinely measure and which require intensive investigation and complex analysis (Rapport et al. 1995). A similar argument could be mounted for other such diffuse ecosystem measures such as “vitality,” “vigor,” and “resilience” (Mageau et al. 1995). Despite their conceptual appeal, these indicators do not lend themselves to routine assessment or the rapid development of possible intervention strategies.

It is important to note, however, that our advocacy of using outbreak data for the purposes outlined in this article does not suggest that such information necessarily should be used as a direct substitute for alternative ecologic measurements. Nor do we imply that conclusions drawn from epidemiologic analysis somehow invalidate those derived from other systems of ecologic monitoring. Many ecologic measures pertain to the health of other (i.e., nonhuman) organisms or systems or may act below the threshold by which the overt appearance of infectious disease in humans may occur. Some environmental agents also operate to cause disease in other or more gradual mechanisms, as in the case of carcinogens or teratogens.

Rather than relying solely on human disease incidence as a bioindicator, we acknowledge that in many situations standard measures of ecosystem health may be entirely synergistic and complementary to outbreak data (Rapport 1999). For example, one sampling strategy that could be successfully integrated with the use of outbreak data is monitoring the abundance and distribution of synanthropes and other organisms that act as intermediaries for human disease. For example, rodent, mosquito, and algal populations not only reflect the potential for transmission, but in themselves may function as integrative indicators of ecosystems function.

Furthermore, there are limitations in the use of outbreak data as a measure of ecosystem disruption that must be recognized. First, communicable disease monitoring and surveillance are clearly less useful options in regions with a low human population density (e.g., circumpolar regions) or where incidence data are erratically obtained, unreliable, or simply not collected. Second, many diseases show underlying variation independent of ecosystem disruption. For example, increases may relate solely to seasonality or other cyclical patterns (e.g., measles outbreaks secondary to human immunity dynamics). Although in such situations the effects of transformed ecosystem may be absent, it is important to consider that ecologic disturbances may also overlie or distort baseline fluctuations (e.g., through acting to enhance “normal” mosquito breeding seasons). As discussed, diseases transmitted principally by direct human-to-human contact (e.g., measles, varicella) are less likely to be affected by ecologic change. However, the degree to which environmental changes contribute or underlie even direct anthropologic disease patterns is becoming increasingly apparent, especially those linked to compromised water supplies and poor sanitation (as illustrated by the relationships between climate change, flooding, and diseases such as cholera and dysentery; e.g., McMichael 1997).

## Conclusion

Burger and Gochfeld (2001) highlight the need for development of bioindicators that can be used for the integrated assessment of both ecologic and human health, and that these must be easily measured and understood, be cost-effective, and have direct societal relevance to gain long-term support. It would appear that human disease incidence meets all of these requirements and, despite certain inherent limitations, can be used for early identification of ecologic disruption.

This process facilitates early intervention, which in turn can decrease the level of “ecosystem distress” and the resultant disease burden in humans. Human disease surveillance pathways could therefore help define areas at ecologic risk (Weinstein et al. 1994). This process would capitalize on an existing health infrastructure that must remain intact in any case if our societies are to maintain the public health gains of the last century. The local, regional, and global levels of our approach will also encourage a renewed perspective of environmental problems. Policy makers and public health officials are often inclined to consider the underlying drivers of ecologic and human health in local or, at best, regional terms. This truncated approach is becoming less and less relevant to current problems. To understand many current environmental health issues, our multilevel approach endorses a move toward a multilevel paradigm, such as those promulgated by the Millennium Ecosystem Assessment (2003) and authors such as Aron and Patz (2001).

The desired goal of the multilevel approach to outbreak data is for both ecologic and human health to be enhanced. This discussion provides further evidence of the undesirability of artificially separating the well-being and viability of communities from that of the biosphere. Outbreak data can act as a pivotal warning system for ecosystem injury and may also be used to guide logical interventions for the simultaneous preservation of ecologic and human health at the local, regional, and global levels. Our recommendation is to acknowledge and exploit the strengths of using human disease surveillance for these purposes.
